# ATF4/MYC Regulates MTHFD2 to Promote NSCLC Progression by Mediating Redox Homeostasis

**DOI:** 10.1155/2022/7527996

**Published:** 2022-08-22

**Authors:** Yixing Gao, Lan Feng, Luping Zhang, Jianhui Geng, Erlong Zhang

**Affiliations:** ^1^Department of Oncology, Xinqiao Hospital, Army Medical University, Chongqing, China; ^2^Key Laboratory of Immunotherapy, Xinqiao Hospital, Army Military Medical University, Chongqing, China; ^3^Institute of Medicine and Equipment for High Altitude Region, College of High Altitude Military Medicine, Army Medical University, Chongqing, China; ^4^Key Laboratory of Extreme Environmental Medicine, Ministry of Education of China, Chongqing, China; ^5^Key Laboratory of High Altitude Medicine, People's Liberation Army, Chongqing, China; ^6^State Key Laboratory of Ultrasound in Medicine and Engineering, Chongqing Medical University, Chongqing, China

## Abstract

**Purpose:**

Methylenetetrahydrofolate dehydrogenase 2 (MTHFD2) has been reported to be overexpressed in non-small-cell lung cancer (NSCLC) and to correlate with malignant proliferation. However, the mechanism of high MTHFD2 expression in NSCLC has not been clarified.

**Methods:**

qPCR, western blot, and immunofluorescence experiments were used to measure the expression of related mRNAs and proteins. Cell apoptosis was measured by flow cytometry and TUNEL assays. The CCK-8 assay was used to determine cell viability. Flow cytometry was used to analyze the cell cycle. ROS, H_2_O_2_, MDA, SOD, and NADPH/NADP^+^ were evaluated by relevant assay kits. Transfection of siRNA or vectors was used to downregulate or upregulate gene expression. Dual-luciferase reporter gene assays were used to evaluate the regulated relationship between MTHFD2 and ATF4 or MYC.

**Results:**

MTHFD2 was highly expressed in NSCLC cells. Knockdown of MTHFD2 inhibited proliferation and increased apoptosis. Furthermore, oxidative factors significantly increased, while antioxidant factors significantly decreased in NSCLC cells with MTHFD2 knockdown, indicating that MTHFD2 was involved in NSCLC progression through the redox pathway. Although MTHFD2 was downregulated with ATF4 silencing, the dual-luciferase reporter assay suggested that ATF4 did not directly mediate MTHFD2 transcription. Further studies revealed that MYC had a transcriptional effect on MTHFD2 and was also regulated by ATF4. PCR, and western blotting experiments with ATF4 knockdown and MYC overexpression as well as ATF4 overexpression and MYC knockdown proved that ATF4 stimulated MTHFD2 through MYC mediation.

**Conclusions:**

ATF4 promoted high expression of MTHFD2 in NSCLC dependent on MYC.

## 1. Background

Metabolic reprogramming has been regarded as a common characteristic of various cancers, and studying its features and regulated mechanism will benefit the interpretation of cancer biological processes and anticancer therapy [1]. Among the most noticeable reprogrammed metabolic pathways in cancer cells, one-carbon metabolism has been greatly involved in cancer initiation and progression [2, 3]. One-carbon metabolism involves cytoplasmic and mitochondrial biosynthetic reactions that bring one-carbon units into various cellular activities. One-carbon units are involved in multiple cellular activities, including nucleotide synthesis, methylation modification, and the production of reducing molecules, which greatly support the malignant phenotype of cancer cells [4]. In recent years, an increasing number of researchers have focused on investigating the changes and roles of the one-carbon pathway in cancer [5, 6]. The role of one-carbon metabolism in cancer has received great attention, and studies targeting one-carbon metabolism for cancer treatment have been well developed.

Among the enzymes mediating one-carbon metabolism, methylenetetrahydrofolate dehydrogenase 2 (MTHFD2) is markedly upregulated in many tumors. Among the 1454 enzymes examined in the work of Nilsson et al., MTHFD2 was the most consistently overexpressed [7]. Moreover, the role of high expression MTHFD2 has been well studied in breast cancer [8–11], acute myeloid leukemia [12–14], lymphoma [15], glioma [16–21], hepatocellular cancer [22], pancreatic cancer [23], renal cell carcinoma [24, 25], colorectal cancer [26–28], lung cancer [29–35], head and neck squamous cell cancer [36, 37], gastric cancer [38], esophageal cancer [39, 40], bladder cancer [41], prostate cancer [42], ovarian cancer [43], and other cancers [44–46]. Upregulated MTHFD2 was reported to support multiple tumor phenotypes, including proliferation [11–16, 20, 24–30, 33–35, 41–43, 45], migration [10, 13, 22, 25, 27, 28, 33, 34], invasion [10, 13, 16, 22, 25], metastasis [26, 34, 43], drug resistance [17, 32, 35, 36, 38, 45], immune evasion [46], metabolic reprogramming [8, 12, 24], self-renewal [30, 32], and poor prognosis of patients [9, 17, 18, 21–23, 25, 26, 31, 33–35, 37, 39, 40, 42].

The expression and function of MTHFD2 in non-small-cell lung cancer (NSCLC) has also been widely reported. MTHFD2 showed high expression in NSCLC cells and tissues from NSCLC patients [29–35]. High MTHFD2 expression promoted lung cancer cell proliferation [29, 30, 33–35] and was significantly correlated with poor prognosis of NSCLC patients [33–35]. This effect of MTHFD2 on lung cancer was closely associated with modulating ROS and NADPH [30]. In addition, another study reported that MTHFD2 sustained the properties of stem cells and gefitinib resistance in lung cancer [32]. Moreover, high expression of circ-MTHFD2 showed clinical significance in the diagnosis, pathological staging, and prognosis of NSCLC [31]. Recently, MTHFD2 was also involved in the character of metastasis [34] and pemetrexed chemoresistance [35] in NSCLC. Based on these studies, we concluded that MTHFD2 played an important role in the development of NSCLC. However, the regulated mechanism of high MTHFD2 expression in NSCLC has not been clarified.

In our work, we investigate the MTHFD2 expression and its role maintaining reductive oxidative homeostasis in NSCLC. Furthermore, we studied the regulated effects of the transcriptional factors ATF4 and MYC on MTHFD2 and found that MYC rather than ATF4 directly mediates MTHFD4 expression. Moreover, our findings also clarified that ATF4 promotes MTHFD4 expression in a MYC-dependent manner.

## 2. Materials and Methods

### 2.1. Cell Culture

Human bronchial epithelial cell lines HBE and BEAS-2B and NSCLC cell lines A549, H358, H1299, and HCC827 were used in this study. These cells were incubated in DMEM (A549, H358, and HCC827) or RPMI-1640 media (HBE, BEAS-2B, and H1299) supplemented with fetal bovine serum (FBS, 10%), L-glutamine (2 mM), penicillin (100 U/ml), and streptomycin (100 *μ*g/ml) at 37°C/5% CO_2_/95% humidity in culture chambers (Thermo Scientific).

### 2.2. qPCR Experiment

cDNA synthesis and qPCR were performed according to our previous protocols [47]. RNA from cells was isolated using the total RNA Kit I (Takara, R6834-02) according to the manufacturer's protocol. cDNA was synthesized using PrimeScript RT reagent kit (Takara, RR047A) with random primers for RT priming. qPCR was performed using SYBR Green (Bio-Rad, RR820A) according to the manufacturer's instructions. The primers used in this study are listed in Table S1.

### 2.3. Western Blot

Whole cell lysates were obtained by resuspending cell pellets in RIPA buffer (Beyotime, P0013E) with a freshly added protease inhibitor tablet (Thermo Scientific, 88265). The whole cell protein extracts (30 *μ*g/well) were separated by 10% SDS-PAGE and then blotted onto polyvinylidene difluoride membranes (BioRad, USA). The membranes were immunoblotted with primary antibodies against *α*-TUBULIN (Proteintech, 11224-1-AP), GAPDH (Proteintech, 10494-1-AP), HSP90 (Proteintech, 60318-1-Ig), MTHFD2 (Proteintech, 12270-1-AP), MYC (Proteintech, 10828-1-AP), and ATF4 (Proteintech, 60035-1-Ig) overnight at 4°C and then additionally incubated with horseradish peroxidase-conjugated secondary antibodies (1 : 2,000; Beyotime, China) at room temperature for 2 h. For quantification, the band intensity of the blot was analyzed by Quantity One software (BioRad, USA).

### 2.4. Immunofluorescence Staining

For the immunofluorescence experiments, cells were cultured in plates for 48 h. Then, the sections from implanted cells were fixed and incubated with primary antibody of MTHFD2 or ATF4, followed by incubation with the corresponding secondary antibodies (Invitrogen, USA). The nuclei were counterstained with 4′, 6-diamidino-2-phenylindole (DAPI, Beyotime, China). The expression of MTHFD2 and ATF4 was observed by confocal fluorescence microscopy.

### 2.5. Cellular Transfection Experiments

Each group of cells was seeded and cultured for 24 hours prior to transfection. Specific small interfering RNA (siRNA) targeting MTHFD2, ATF4, and MYC (Table S2) or scrambled siRNA (the final concentration of siRNA was 20 nM) from GenePharma (China) or ATF4 and MYC vectors from Vector Builder (China) were transfected into the cells using Lipofectamine RNAiMAX or Lipofectamine 3000 (Invitrogen, USA) according to the manufacturer's instructions. The cells were collected after 72 h.

### 2.6. Cell Growth Assay

Cells were plated in 96-well plates (5000 cells per well with 100 *μ*l of growth medium) and transfected 24 h later. Cell viability was determined 72 h after transfection. The cell viability assay was carried out with the Cell Counting Kit-8 (CCK-8, Dojindo, Kumamoto, Japan) according to the manufacturer's instructions and determined at 450 nm by a 96-well plate spectrophotometer (Multiskan GO, Thermo Scientific, USA).

### 2.7. Cell Cycle Assay

Cell cycle analysis was performed using an Accuri 6 flow cytometer (Accuri Cytometers, Inc., Ann Arbor, MI, USA) and Cell Quest software following the manufacturer's protocols. Cells were collected, centrifuged, and fixed with 70% ethanol. The samples were analyzed by flow cytometry.

### 2.8. Cell Apoptosis Assay

Cell apoptosis was measured by an Annexin V-fluorescein isothiocyanate apoptosis detection kit (Keygen Biotech, Nanjing, China). Cells were collected, centrifuged, washed with phosphate-buffered saline, and counted with an electronic cytometer (Beckman Coulter, Miami, FL). Approximately 1.0 × 10^5^ cells were resuspended in 190 *μ*l of Annexin V-fluorescein isothiocyanate-binding buffer, and subsequently, 5 *μ*l of Annexin V-fluorescein isothiocyanate and 5 *μ*l of propidium iodide were added and incubated for 10 min with the samples in the dark at room temperature. The fluorescence of the cells was detected, and the results were analyzed by flow cytometry.

### 2.9. TUNEL Assay

Sections from implanted cells were fixed in 4% paraformaldehyde, permeabilized in methanol, and stained using a TdT-mediated dUTP nick-end labeling (TUNEL) reaction mixture. Apoptotic cells in sections were determined with an in situ cell death detection kit (Roche) according to the manufacturer's instructions.

### 2.10. Determination of ROS, H_2_O_2_, MDA, SOD, and NADPH/NAPD^+^ Levels

The levels of cellular ROS were determined by flow cytometry using a ROS assay kit (S0033, Beyotime, China). The levels of cellular H_2_O_2_, MDA, SOD, and NADPH/NAPD^+^ were determined by a microplate reader using Beyotime assay kits (S0038, S0131S, S0101S, and S0179).

### 2.11. Luciferase Promoter Assay

Cells were plated into a 24-well plate to achieve 50% confluence on the day of transfection. A dual luciferase reporter assay system (Promega, Madison, USA) was used according to the manufacturer's protocol. Briefly, a mixture containing Lipofectamine LTX reagent (Invitrogen, Carlsbad, USA), luciferase MTHFD2 promoter (~2000 bp upstream of the start site) vector, and vector control or ATF4 (MYC) vector (Vector Builder, China) was added to each well. Luciferase and Renilla signals were measured 72 h after transfection according to the recommended protocol.

### 2.12. Statistical Analysis

Statistical analysis was carried out using SPSS 19.0 software. The experiments were analyzed using independent *t*-tests. Data are presented as the mean ± SD, and a value of *p* < 0.05 was assumed to indicate a statistically significant result. All experiments were independently carried out in triplicate.

## 3. Results

### 3.1. Increased MTHFD2 Expression in NSCLC Cells

**Figure 1:**
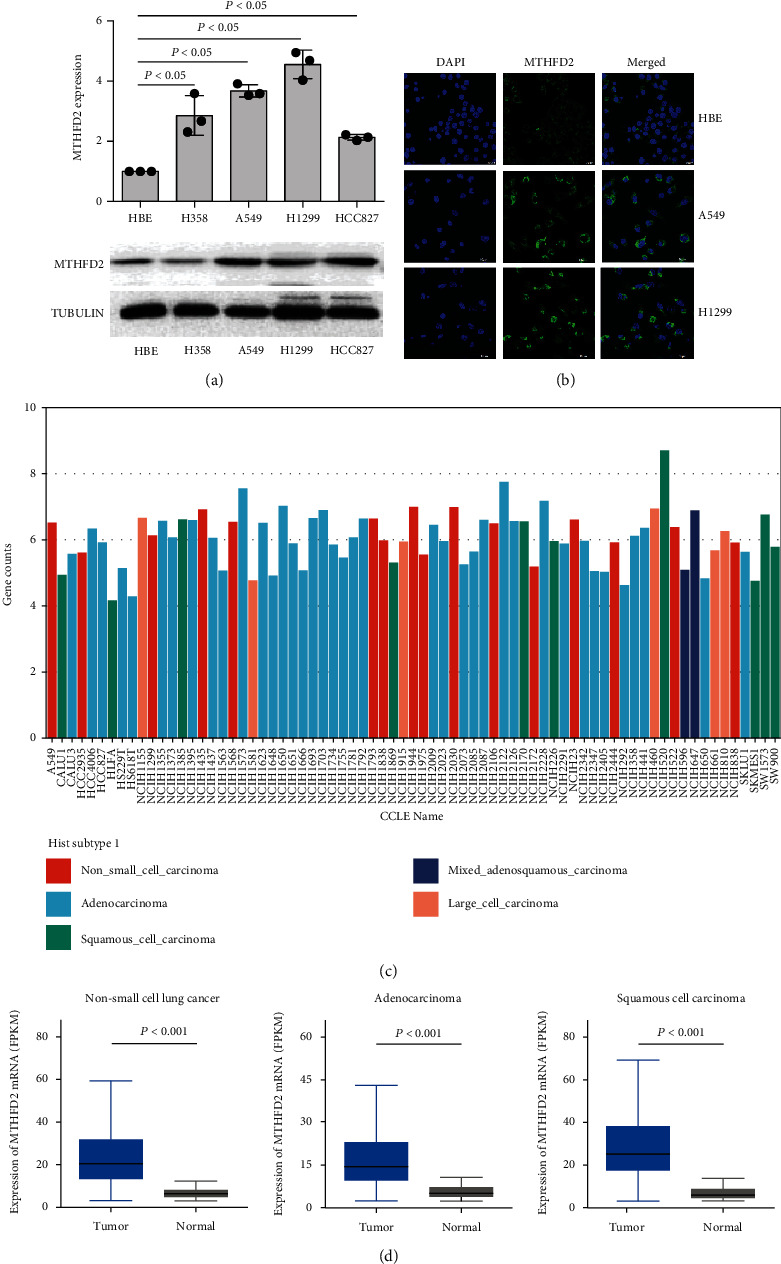
MTHFD2 expression in NSCLC cell lines (a) mRNA and protein levels of MTHFD2 in HBE, H358, A549, H1299, and HCC827 cells (compared to HBE cells). (b) Immunofluorescence observation of MTHFD2 expression in HBE, A549, and H1299 cells. All the data were from three individual tests. Statistical analyses between groups were performed with ANOVA followed by LSD post hoc test (^∗^*p* < 0.05, compared with HBE cells). (c) Heat map of MTHFD2 expression levels among NSCLC cell lines from the CCLE database. (d) The expression of MTHFD2 mRNA levels in tumor and normal tissues of NSCLC patients from the TCGA database.

To investigate the role of MTHFD2 in non-small-cell lung cancer, we firstly evaluated the expression of MTHFD2 in several NSCLC cell lines. As shown in Figure 1(a), A549, H1299, and HCC827 cells showed higher MTHFD2 mRNA and protein levels than HBE cells. The expression of MTHFD2 in NSCLC cells also showed similar results compared to BEAS-2B cells (Figure S1), consistent with previous work [29]. Moreover, the immunofluorescence assay also proved that A549 and H1299 cells displayed increased MTHFD2 expression compared with HBE cells (Figure 1(b)). Noticeably, the CCLE database showed common expression of MTHFD2 in NSCLC cells (Figure 1(c)), implying that MTHFD2 is a signature among NSCLC cells. Furthermore, the overexpression of MTHFD2 in NSCLC (including adenocarcinoma and squamous cell carcinoma) was also supported by The Cancer Genome Atlas database (Figure 1(d)). These results demonstrate that MTHFD2 is overexpressed in NSCLC cells.

### 3.2. Effects of MTHFD2 Silencing on the Biological Activities of NSCLC Cells

**Figure 2:**
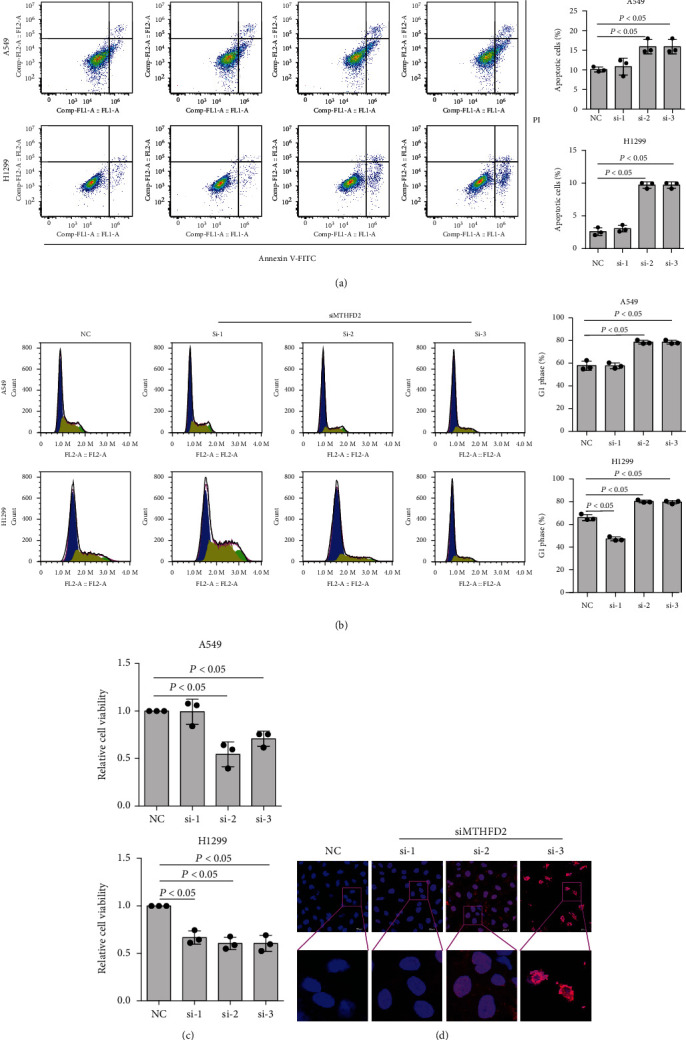
Effect of MTHFD2 knockdown on cell viability, cell cycle, and apoptosis in A549 and H1299 cells. Percentage of apoptotic cells (a), percentage of G1 phase (b), and cell viability (c) in A549 and H1299 cells and TUNEL assays of A549 cells (d) were performed under MTHFD2 knockdown. All the data were from three individual tests. Statistical analyses between groups were performed with ANOVA followed by LSD post hoc test (^∗^*p* < 0.05, compared with the negative control (NC)).

To evaluate the function of MTHFD2 in NSCLC, we established MTHFD2-targeted specific siRNAs and measured their inhibition rates (Figure S2). Then, we performed experiments to observe the effect of MTHFD2 on cell viability, cell cycle, and apoptosis. The results of flow cytometry and TUNEL assays showed that MTHFD2 knockdown could promote the apoptosis of NSCLC cells (Figures 2(a) and 2(d)). Moreover, MTHFD2 silencing increased the percentage of NSCLC cells in the G1 phase and arrested cell proliferation (Figure 2(b)). In addition, the CCK-8 experiments indicated that knockdown of MTHFD2 inhibited the growth of A549 and H1299 cells (Figure 2(c)). These results suggest that MTHFD2 plays an important role in maintaining NSCLC proliferation.

### 3.3. MTHFD2 Is Involved in Regulating NSCLC Redox Homeostasis

**Figure 3:**
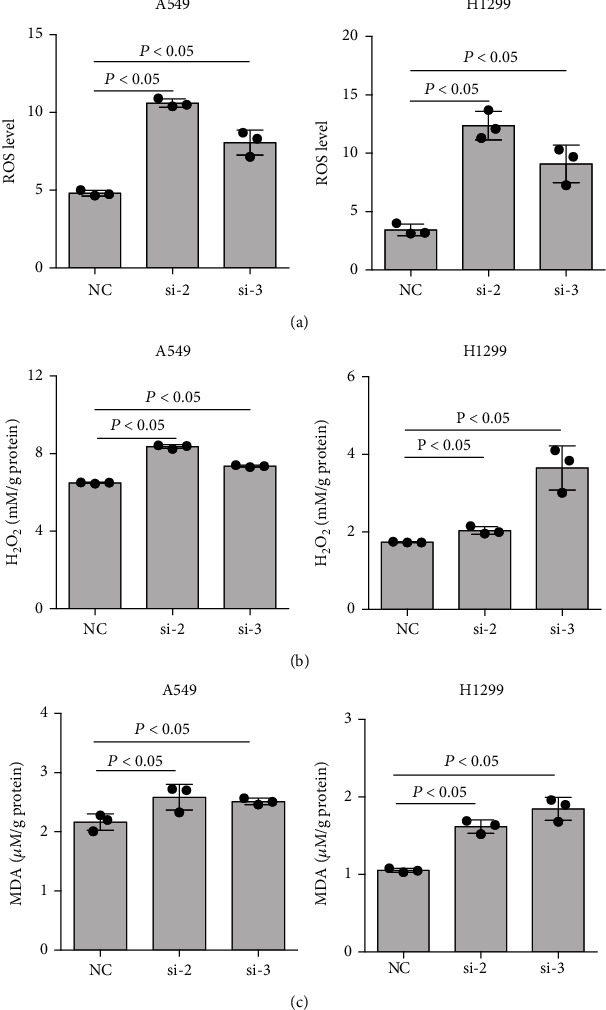
Effect of MTHFD2 knockdown on oxidative factors in A549 and H1299 cells. The levels of ROS (a), H_2_O_2_ (b), and MDA (c) in A549 and H1299 cells were determined under MTHFD2 knockdown. All the data were from three individual tests. Statistical analyses between groups were performed with ANOVA followed by LSD post hoc test (^∗^*p* < 0.05, compared with the negative control (NC)).

**Figure 4:**
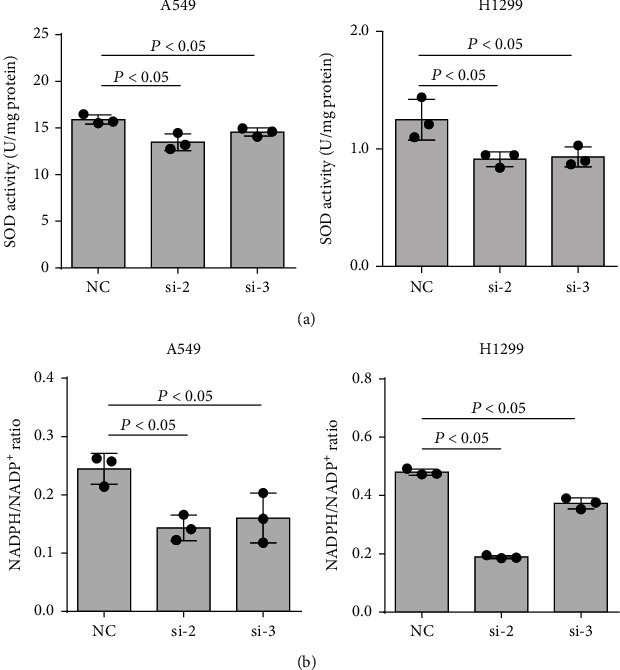
Effect of MTHFD2 knockdown on antioxidative factors in A549 and H1299 cells. The levels of SOD (a) and NADPH/NADP^+^ (b) in A549 and H1299 cells were determined under MTHFD2 knockdown. All the data were from three individual tests. Statistical analyses between groups were performed with ANOVA followed by LSD post hoc test (^∗^*p* < 0.05, compared with the negative control (NC)).

Given the results above, we investigated the mechanism by which MTHFD2 supported NSCLC progression. Because the physiological function of MTHFD2 is catalyzing 5,10-methyl-lenetetrahydrofolate to 10-formyltetrahydrofolate along with NAD(P)^+^ to NAD(P)H, we proposed that MTHFD2 could promote NSCLC progression by regulating reductive/oxidative (redox) processes. With MTHFD2 knockdown, we observed that oxidative factors, including ROS, H_2_O_2_, and MDA, significantly increased (Figure 3). Conversely, antioxidative factors, including SOD and NADPH/NADP^+^, showed a marked reduction in NSCLC cells (Figure 4). These results indicate that high MTHFD2 expression promotes antioxidative capacity in NSCLC.

### 3.4. ATF4 Indirectly Mediates MTHFD2 Expression in NSCLC

**Figure 5:**
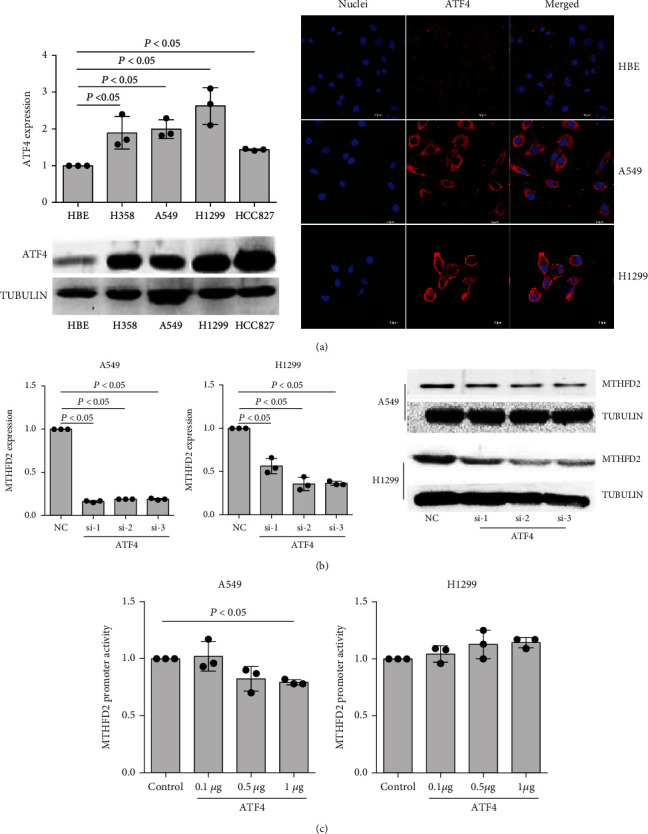
The regulated effect of ATF4 on MTHFD2 in A549 and H1299 cells. (a) mRNA and protein levels of ATF4 in HBE, H358, A549, H1299, and HCC827 cells (compared to HBE cells). Immunofluorescence observation of ATF4 expression in HBE, A549 and H1299 cells. (b) Levels of MTHFD2 mRNA and protein in A549 and H1299 cells with ATF4 knockdown (compared to NC). (c) Relative MTHFD2 luciferase promoter activity with ATF4 overexpression in A549 and H1299 cells (compared to the control vector). ^∗^*p* < 0.05.

Based on the high expression of MTHFD2 in NSCLC, we clarified the regulated process of this characteristic in following. From a previous report, we inferred that ATF4 could regulate the expression of MTHFD2 [48]. Then, the expression levels of ATF4 in NSCLC cells were detected, and the results of qPCR, western blotting, and immunofluorescence experiments showed that these cells displayed significantly higher expression than HBE cells (Figure 5(a)). Knockdown of ATF4 induced a noticeable decrease in MTHFD2 expression in A549 and H1299 cells (Figure 5(b) and Figure S3). However, MTHFD2 luciferase promoter activity did not exhibit a significant change with ATF4 vector transfection (Figure 5(c)). These results imply that ATF4 indirectly, but not directly, promotes MTHFD2 in NSCLC.

### 3.5. MYC Transcriptionally Regulates MTHFD2 Expression in NSCLC

**Figure 6:**
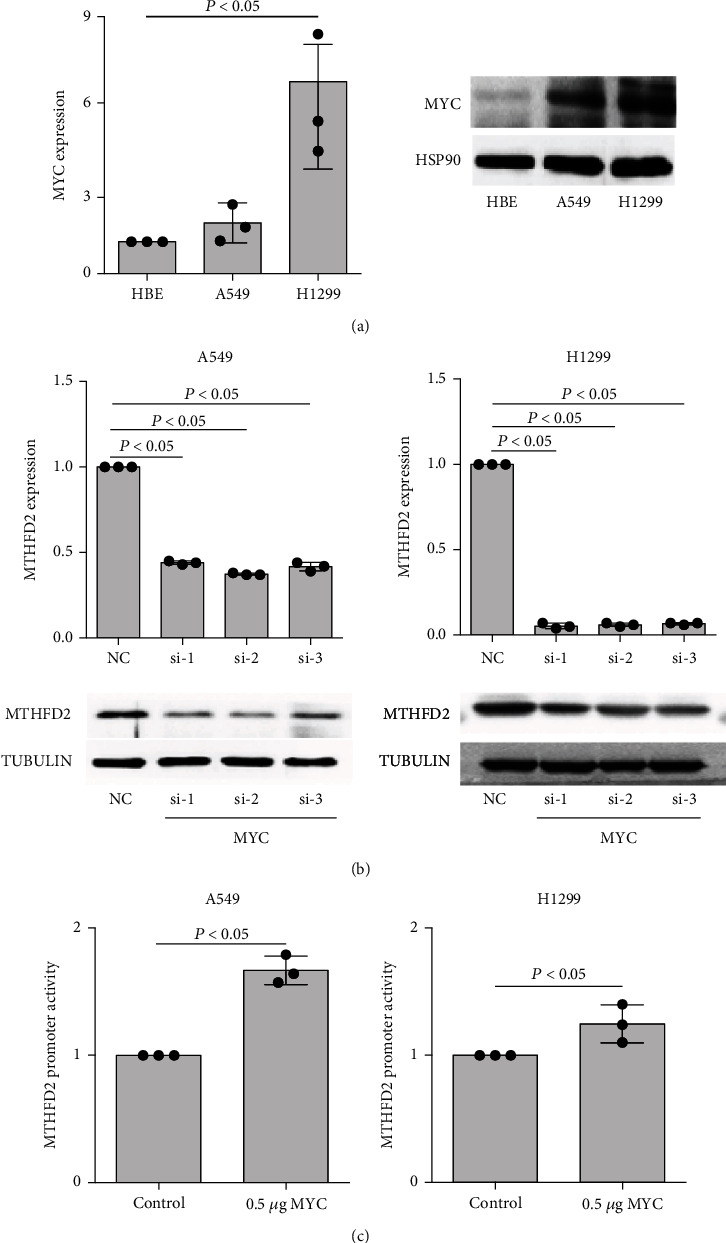
The regulated effect of MYC on MTHFD2 in A549 and H1299 cells. (a) mRNA and protein levels of MYC in BEAS-2B, A549, and H1299 cells (compared to BEAS-2B cells). (b) Levels of MTHFD2 mRNA and protein in A549 and H1299 cells with MYC knockdown (compared to NC). (c) Relative MTHFD2 luciferase promoter activity with MYC overexpression in A549 and H1299 cells (compared to the control vector). ^∗^*p* < 0.05. All the data were from three individual tests. Statistical analyses between groups were performed with ANOVA followed by LSD post hoc test.

Recent studies reported that MYC mediated MTHFD2 transcriptional expression in tumors [12, 26], and we further investigated the regulated role of MYC on MTHFD2 expression in NSCLC. Compared with HBE cells, A549 and H1299 cells presented distinctly higher levels of MYC (Figure 6(a)). MTHFD2 significantly decreased under MYC silencing in A549 and H1299 cells (Figure 6(b) and Figure S4). Furthermore, MTHFD2 luciferase promoter activity displayed a significant increase with MYC vector transfection (Figure 6(c)). These results indicate that MYC transcriptionally regulates MTHFD2 expression in NSCLC.

### 3.6. ATF4 Regulates MTHFD2 Expression through MYC in NSCLC

**Figure 7:**
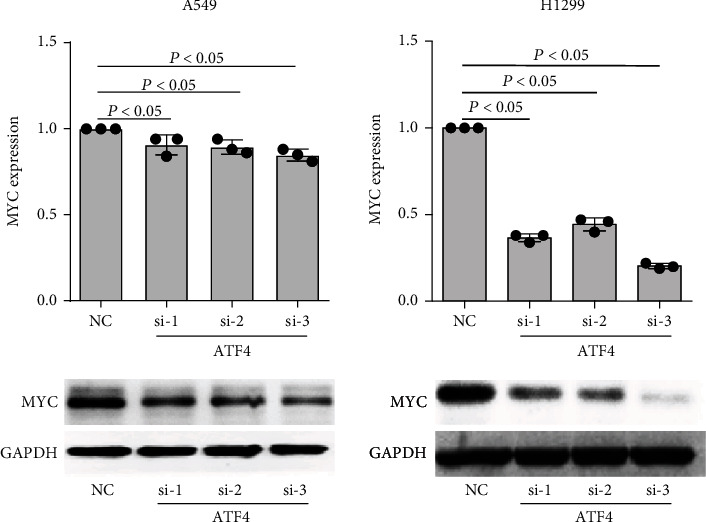
The regulated effect of ATF4 on MYC in A549 and H1299 cells. Levels of MYC mRNA and protein in A549 and H1299 cells with ATF4 knockdown. All the data were from three individual tests. Statistical analyses between groups were performed with ANOVA followed by LSD post hoc test (^∗^*p* < 0.05, compared to NC).

From the results above, we inferred that both ATF4 and MYC are involved in MTHFD2 expression in NSCLC. However, the relationship between ATF4 and MYC in NSCLC has not been reported. Therefore, we observed the expression of MYC with ATF4 knockdown in A549 and H1299 cells. The result showed that MYC was downregulated with ATF4 silencing (Figure 7).

**Figure 8:**
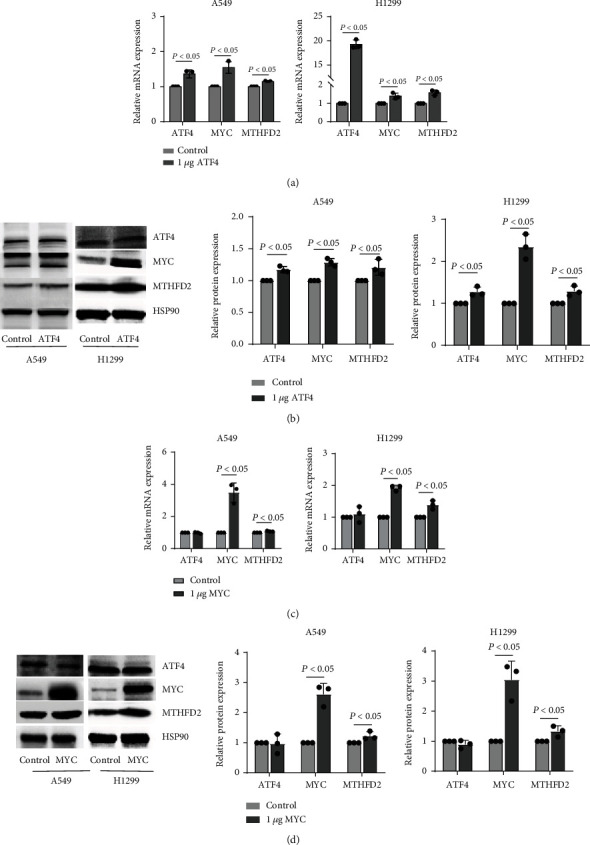
Expression of ATF4, MYC and MTHFD2 in A549 and H1299 cells with overexpression ATF4 or MYC. mRNA (a) and protein (b) expression of ATF4, MYC, and MTHFD2 after transfection with 1 *μ*g ATF4 vector. mRNA (c) and protein (d) expression of ATF4, MYC, and MTHFD2 after transfection with 1 *μ*g MYC vector. ^∗^*p* < 0.05, compared to the control (control vector). All the data were from three individual tests. Statistical analyses between groups were performed with ANOVA followed by LSD post hoc test.

**Figure 9:**
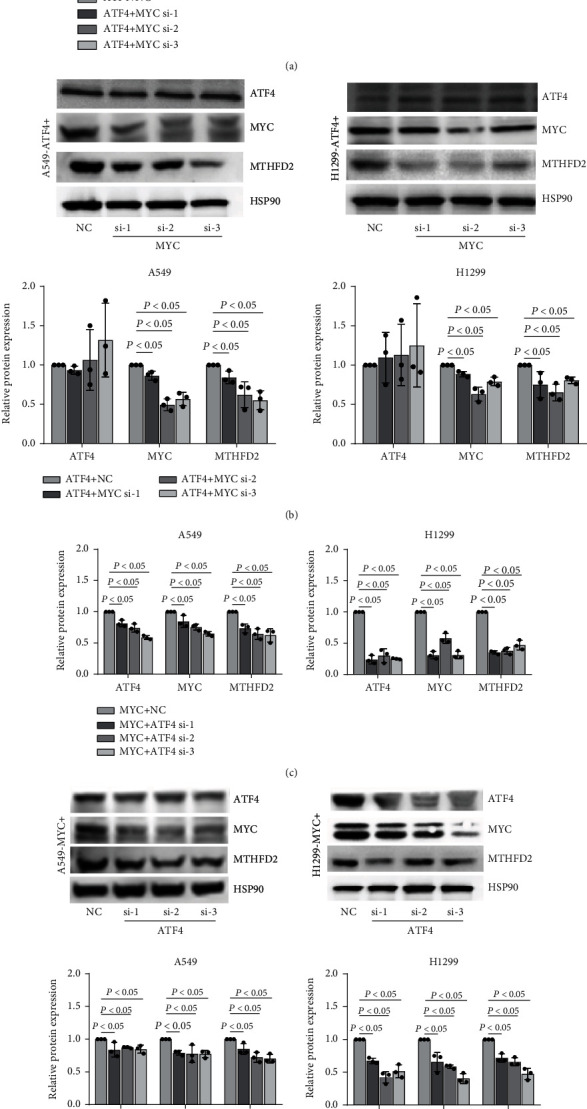
The regulated effect of ATF4 and MYC on MTHFD2 in A549 and H1299 cells. mRNA (a) and protein (b) expression of ATF4, MYC, and MTHFD2 in A549 and H1299 cells transfected with 1 *μ*g ATF4 vector and MYC knockdown (compared to the ATF4 vector and siRNA negative control groups). mRNA (c) and protein (d) expression of ATF4, MYC, and MTHFD2 in A549 and H1299 cells transfected with 1 *μ*g MYC vector and ATF4 knockdown (compared to the MYC vector and siRNA negative control groups). ^∗^*p* < 0.05. All the data were from three individual tests. Statistical analyses between groups were performed with ANOVA followed by LSD post hoc test.

To investigate whether ATF4 promoted MTHFD2 through MYC in NSCLC, we transfected ATF4 vectors and MYC vectors simultaneously into A549 and H1299 cells and observed the expression levels of ATF4, MYC, and MTHFD2. The results indicated that ATF4, MYC, and MTHFD2 were upregulated under ATF4 vector transfection (Figures 8(a) and 8(b)). And the expression of MYC and MTHFD2 without ATF4 showed marked increase under MYC vector transfection (Figures 8(c) and 8(d)). These results indicate that ATF4 upregulates MTHFD2 by MYC. To further validate the regulated process, we transfected ATF4 vectors with MYC knockdown and MYC vectors with ATF4 knockdown simultaneously in A549 and H1299 cells and detected the expression levels of ATF4, MYC, and MTHFD2. The results indicated that MYC and MTHFD2 without ATF4 were downregulated under ATF4 vector transfection with MYC knockdown (Figures 9(a) and 9(b)). The expression of ATF4, MYC, and MTHFD2 all showed marked decrease under MYC vector transfection and ATF4 knockdown (Figures 9(c) and 9(d)). These experiments proposed that ATF4-regulated MTHFD2 expression is dependent on MYC in NSCLC.

## 4. Discussion

MTHFD2 is a key enzyme in mitochondrial one-carbon metabolism and promotes the conversion of 5,10-methylenetetrahyderofolate to 10-formyl-tetrahydrofolate [49]. During this process, the one-carbon formyl groups are substrates for nucleotide synthesis, and NADPH generated from NADP^+^ displays antioxidative ability, which could promote malignant tumor progression. Moreover, MTHFD2 could induce oncogene expression by regulating DNA replication, RNA translation, and epigenetic modification [24, 50]. Multiple studies mentioned above support the hypothesis that MTHFD2 facilitates the initiation and progression of diverse original tumors by these pathways, which was summarized by Zhu and Leung [51]. Therefore, MTHFD2 has been widely recognized as a potential therapeutic target for tumor treatment [52, 53]. In recent years, several groups have developed studies of MTHFD2 inhibitors, some of which have presented potential antitumor efficacy in experiments in vitro and in vivo [54–58].

The role of one-carbon metabolism was firstly reported in NSCLC in 2012 [59]. Previous studies and our experiments have proven the high expression and tumor-promoting effect of MTHFD2 in NSCLC [29–35]. And in our study, silence of MTHFD2 increased oxidative capacity and decreased antioxidative capacity, which could induce cellular damage and growth inhibition, which indicated that knockdown of MTHFD2 could promote cell cycle arrest by increasing oxidative factors.

**Figure 10:**
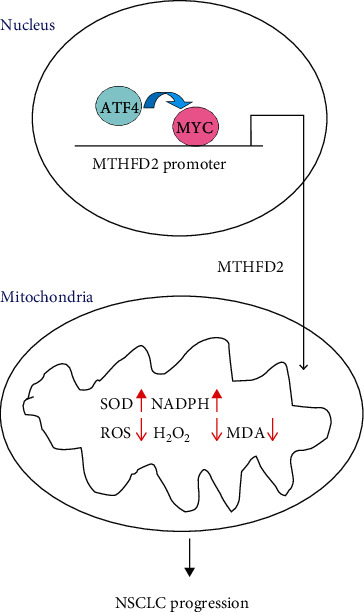
MTHFD2 overexpression mediated by ATF4/MYC promoted NSCLC progression by maintaining oxidative balance.

Moreover, the potential regulated mechanism of MTHFD2 expression in NSCLC has not been explained. mTOR complex I (mTORC1) is a central coordinator of metabolic processes [60], and Ben-Sahra et al. demonstrated that mTORC1 regulated MTHFD2 expression dependent on activating transcription factor 4 (ATF4) in normal and cancer cells [48]. They hypothesized that ATF4 could directly regulate MTHFD2 expression. However, our experiments indicated that MTHFD2 was indeed adjusted by ATF4 but not directly. Moreover, the transcription factor MYC is a master regulator of cell metabolism. Our study suggested that MYC could bind to the promoter region of MTHFD2 and transcriptionally regulate MTHFD2 in NSCLC, consistent with previous experiments in acute myeloid leukemia [12] and colorectal cancer [26]. Furthermore, the results of our experiments also found that MYC could be downregulated under ATF4 knockdown and that ATF4 regulated MTHFD2 expression through MYC instead of direct mediation (Figure 10). In addition, miR-30a-3p was recently reported to target MTHFD2 in small cell lung cancer [61]. Although miR-30a-3p showed increased expression in NSCLC, it did not participate in MTHFD2 regulation (data not shown).

## 5. Conclusion

High expression of MTHFD2 was identified to be involved NSCLC progression by regulating redox homeostasis. Moreover, ATF4/MYC regulated high MTHFD2 expression in NSCLC, which provides a comprehensive understanding of MTHFD2 in NSCLC.

## Data Availability

The datasets used and/or analyzed during the current study are available from the corresponding author on reasonable request.

## Supplementary Materials

Supplementary 1 Figure S1: mRNA and protein levels of MTHFD2 in BEAS-2B, H358, A549, H1299, and HCC827 cells. All the data were from three individual tests. Statistical analyses between groups were performed with ANOVA followed by LSD post hoc test (^∗^*p* < 0.05, compared with BEAS-2B cells).

Supplementary 2 Figure S2: levels of MTHFD2 mRNA and protein in A549 and H1299 cells with MTHFD2 knockdown by siRNA. All the data were from three individual tests. Statistical analyses between groups were performed with ANOVA followed by LSD post hoc test (^∗^*p* < 0.05, compared with the negative control (NC)).

Supplementary 3 Figure S3: levels of ATF4 mRNA and protein in A549 and H1299 cells with ATF4 knockdown by siRNA. All the data were from three individual tests. Statistical analyses between groups were performed with ANOVA followed by LSD post hoc test (^∗^*p* < 0.05, compared with the negative control (NC)).

Supplementary 4 Figure S4: levels of MYC mRNA and protein in A549 and H1299 cells with MYC knockdown by siRNA. All the data were from three individual tests. Statistical analyses between groups were performed with ANOVA followed by LSD post hoc test (^∗^*p* < 0.05, compared with the negative control (NC)).

Supplementary 5 Table S1: sequences of primers used for qPCR. Table S2: the sequences of small interfering RNA (siRNA).
