# Supplementary figures and images for: ATF4/MYC Regulates MTHFD2 to Promote NSCLC Progression by Mediating Redox Homeostasis
Source: Dis Markers. 2022 Aug 22;2022:7527996. doi: 10.1155/2022/7527996 (PMC9425107; doi:10.1155/2022/7527996)

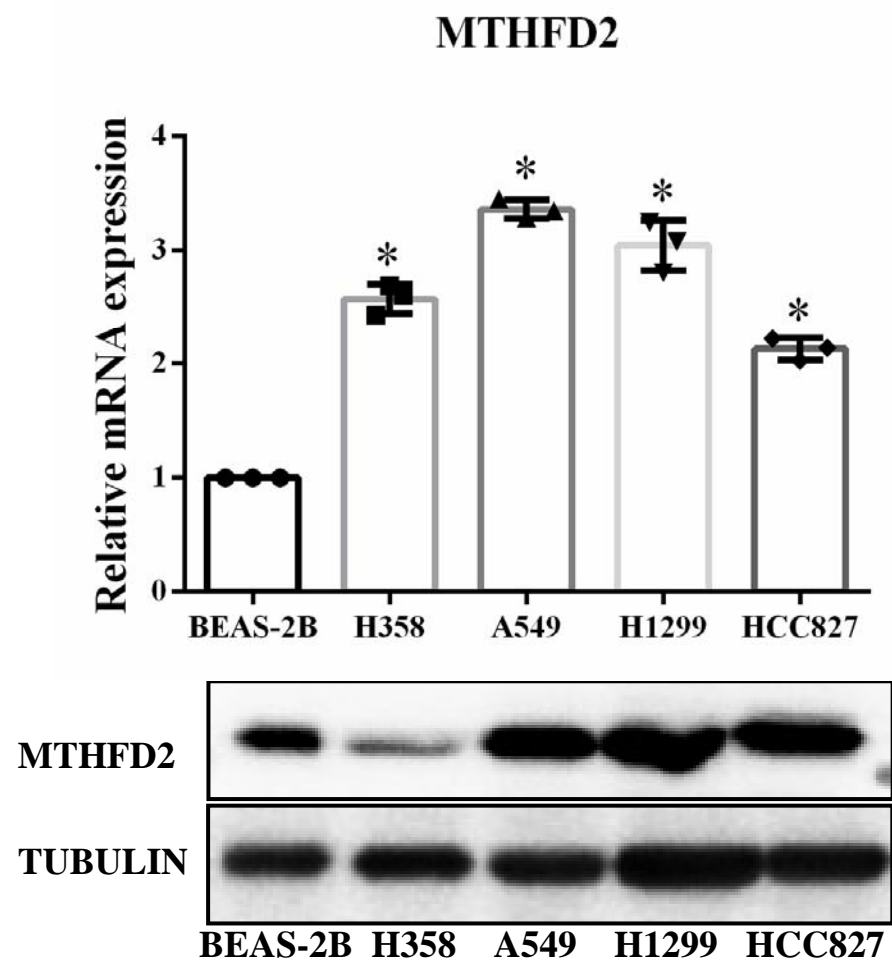

Figure S1

Supplement: Supplementary 1 — Figure S1: mRNA and protein levels of MTHFD2 in BEAS-2B, H358, A549, H1299, and HCC827 cells. All the data were from three individual tests. Statistical analyses between groups were performed with ANOVA followed by LSD post hoc test (∗p < 0.05, compared with BEAS-2B cells). [file 7527996.f1.pdf]

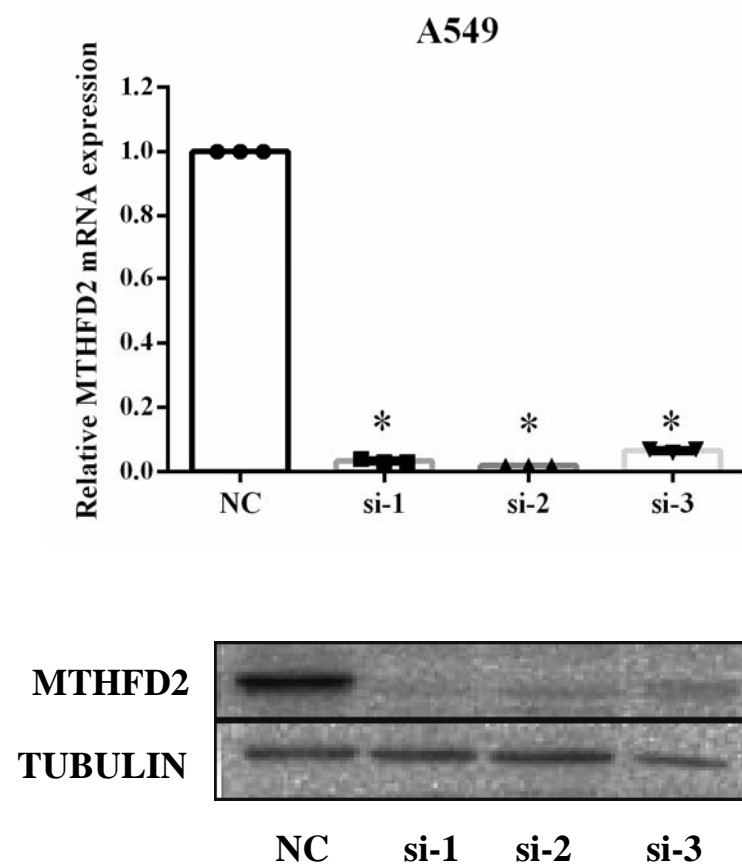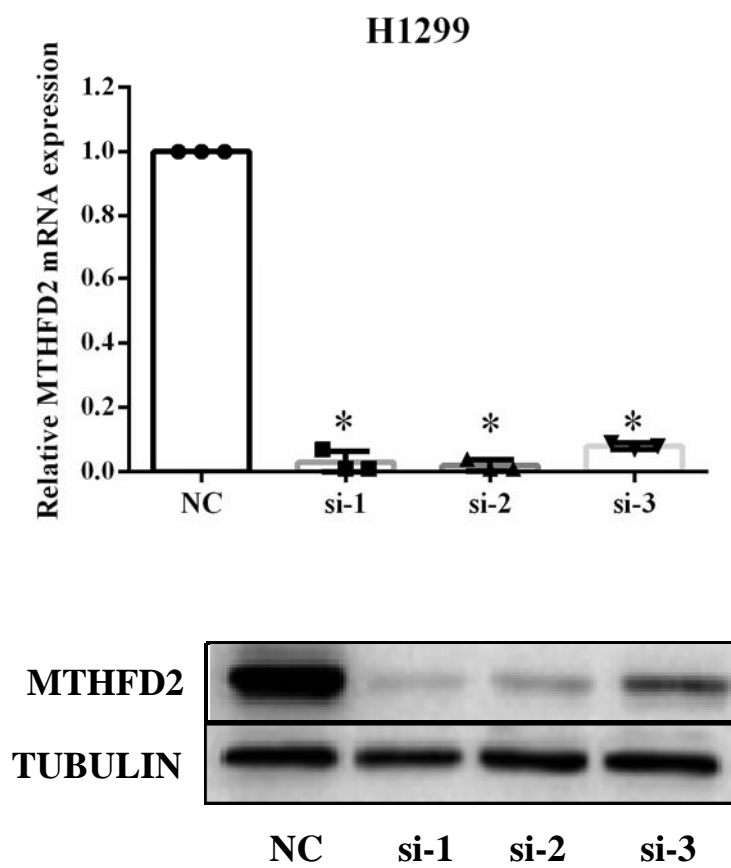

Figure S2

Supplement: Supplementary 2 — Figure S2: levels of MTHFD2 mRNA and protein in A549 and H1299 cells with MTHFD2 knockdown by siRNA. All the data were from three individual tests. Statistical analyses between groups were performed with ANOVA followed by LSD post hoc test (∗p < 0.05, compared with the negative control (NC)). [file 7527996.f2.pdf]

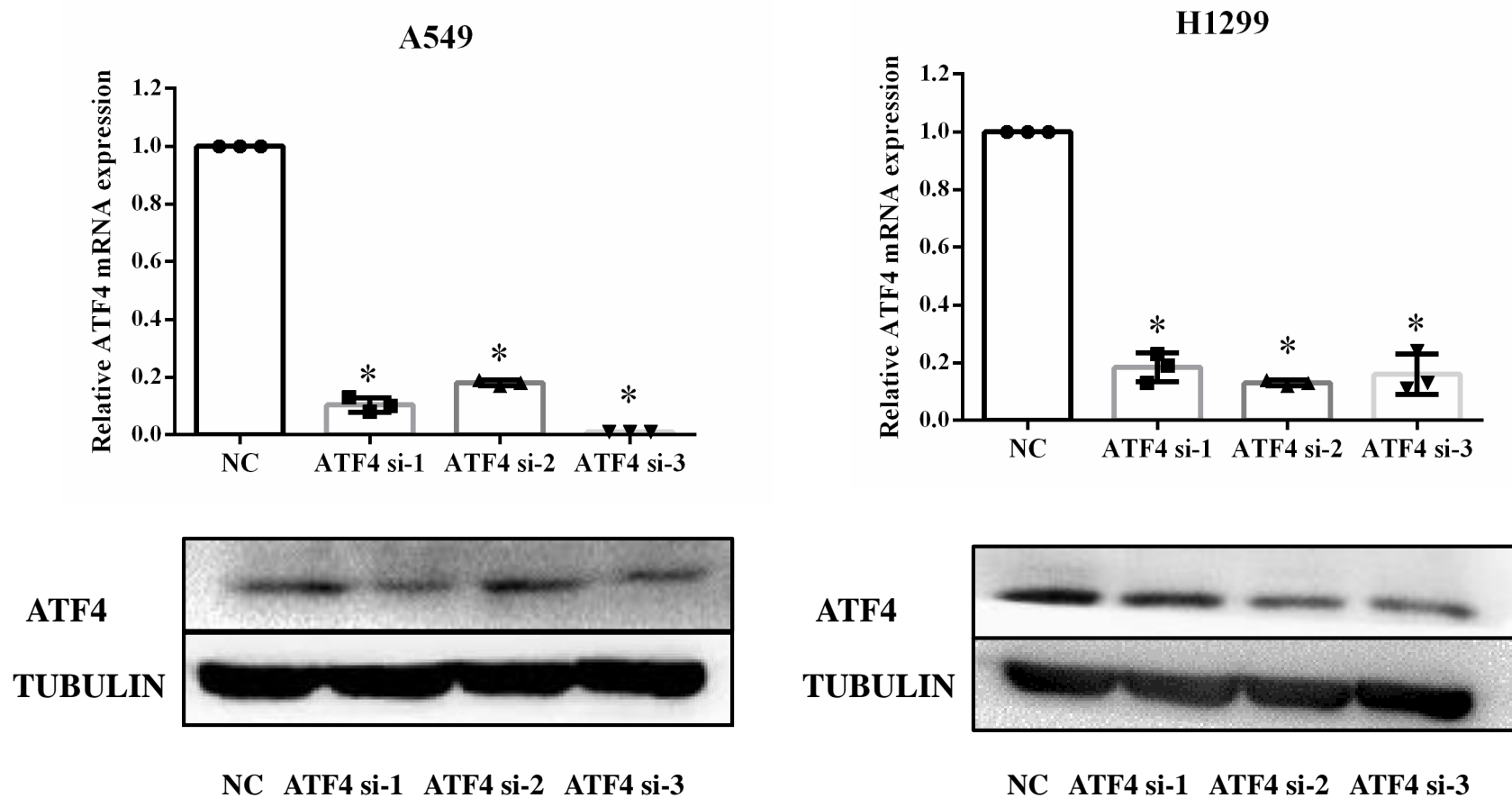

Figure S3

Supplement: Supplementary 3 — Figure S3: levels of ATF4 mRNA and protein in A549 and H1299 cells with ATF4 knockdown by siRNA. All the data were from three individual tests. Statistical analyses between groups were performed with ANOVA followed by LSD post hoc test (∗p < 0.05, compared with the negative control (NC)). [file 7527996.f3.pdf]

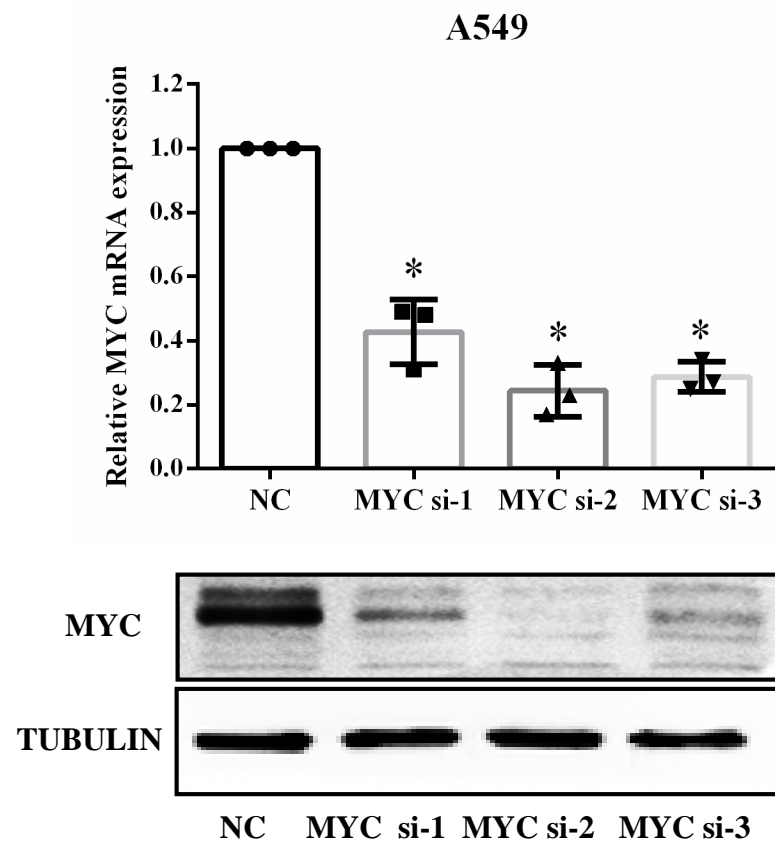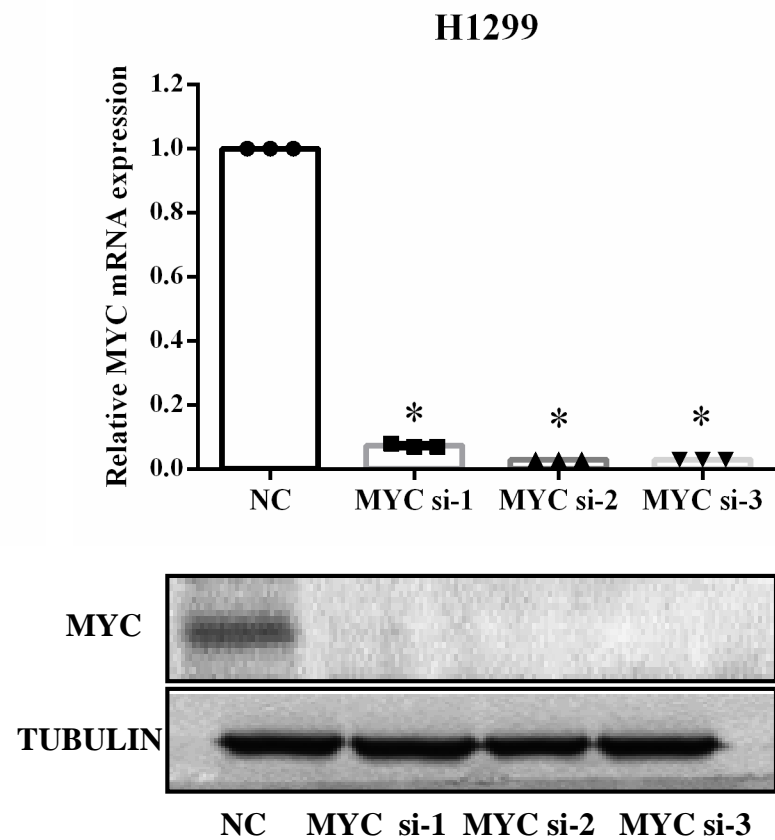

Figure S4

Supplement: Supplementary 4 — Figure S4: levels of MYC mRNA and protein in A549 and H1299 cells with MYC knockdown by siRNA. All the data were from three individual tests. Statistical analyses between groups were performed with ANOVA followed by LSD post hoc test (∗p < 0.05, compared with the negative control (NC)). [file 7527996.f4.pdf]
