# Supplementary material for: ATF4/MYC Regulates MTHFD2 to Promote NSCLC Progression by Mediating Redox Homeostasis
Source: Dis Markers. 2022 Aug 22;2022:7527996. doi: 10.1155/2022/7527996 (PMC9425107; doi:10.1155/2022/7527996)
Supplement: Supplementary 5 — Table S1: sequences of primers used for qPCR. Table S2: the sequences of small interfering RNA (siRNA). [file 7527996.f5.docx]

Table S1. Sequences of primers used for qPCR.

| **Gene** | **Forward primer (5′‒3′)** | **Reverse primer(5′‒3′)** |
| --- | --- | --- |
| MTHFD2 | GATCCTGGTTGGCGAGAATCC | TCTGGAAGAGGCAACTGAACA |
| ATF4 | ATGACCGAAATGAGCTTCCTG | GCTGGAGAACCCATGAGGT |
| MYC | GGCTCCTGGCAAAAGGTCA | CTGCGTAGTTGTGCTGATGT |
| ACTIN | CATGTACGTTGCTATCCAGGC | CTCCTTAATGTCACGCACGAT |

Table S2. The sequences of small interfering RNA (siRNA).

| **Target Gene** | **siRNA** | **Sense (5′‒3′)** | **Antisense (5′‒3′)** |
| --- | --- | --- | --- |
| MTHFD2 | siRNA-1 | GCUGCGACUUCUCUAAUGUTT | ACAUUAGAGAAGUCGCAGCTT |
|  | siRNA-2 | GGAAUCAACAGUGAGACAATT | UUGUCUCACUGUUGAUUCCTT |
|  | siRNA-3 | CCUUGUUCAGUUGCCUCUUTT | AAGAGGCAACUGAACAAGGTT |
| ATF4 | siRNA-1 | GGGUAUAGAUGACCUGGAATT | UUCCAGGUCAUCUAUACCCTT |
|  | siRNA-2 | CCACUCCAGAUCAUUCCUUTT | AAGGAAUGAUCUGGAGUGGTT |
|  | siRNA-3 | CCUGAAAGAUUUGAUAGAATT | UUCUAUCAAAUCUUUCAGGTT |
| MYC | siRNA-1 | GAGGAUAUCUGGAAGAAAUTT | AUUUCUUCCAGAUAUCCUCTT |
|  | siRNA-2 | GCUUGUACCUGCAGGAUCUTT | AGAUCCUGCAGGUACAAGCTT |
|  | siRNA-3 | GGAAGAAAUCGAUGUUGUUTT | AACAACAUCGAUUUCUUCCTT |
